# Generalization Mediates Sensitivity to Complex Odor Features in the Honeybee

**DOI:** 10.1371/journal.pone.0001704

**Published:** 2008-02-27

**Authors:** Geraldine A. Wright, Sonya M. Kottcamp, Mitchell G. A. Thomson

**Affiliations:** 1 Biology, Newcastle University, Newcastle upon Tyne, United Kingdom; 2 Rothenbuhler Honeybee Laboratory, Ohio State University, Columbus, Ohio, United States of America; University of Edinburgh, United Kingdom

## Abstract

Animals use odors as signals for mate, kin, and food recognition, a strategy which appears ubiquitous and successful despite the high intrinsic variability of naturally-occurring odor quantities. Stimulus generalization, or the ability to decide that two objects, though readily distinguishable, are similar enough to afford the same consequence [1], could help animals adjust to variation in odor signals without losing sensitivity to key inter-stimulus differences. The present study was designed to investigate whether an animal's ability to generalize learned associations to novel odors can be influenced by the nature of the associated outcome. We use a classical conditioning paradigm for studying olfactory learning in honeybees [2] to show that honeybees conditioned on either a fixed- or variable-proportion binary odor mixture generalize learned responses to novel proportions of the same mixture even when inter-odor differences are substantial. We also show that the resulting olfactory generalization gradients depend critically on both the nature of the stimulus-reward paradigm and the intrinsic variability of the conditioned stimulus. The reward dependency we observe must be cognitive rather than perceptual in nature, and we argue that outcome-dependent generalization is necessary for maintaining sensitivity to inter-odor differences in complex olfactory scenes.

## Introduction

Natural odors are typically composed of multiple volatile compounds, each with its own concentration and molecular identity. Both quantitative and qualitative aspects of odor composition appear, however, to be subject to a high degree of variability even for odors emitted from the same source [3], [4]. This presents a considerable challenge to potential receivers of odor signals: how is an animal to maintain sensitivity to relevant inter-odor differences in spite of such naturally-occurring heterogeneity in odor composition? Sensitivity to irrelevant inter-odor differences could make it impossible for an animal to recognize an odor and respond appropriately. Yet, failing to discriminate subtle differences amongst odors would be a handicap if such differences were relevant to the outcomes associated with odors [5]–[8]. Since this dilemma is faced by all animals that use odors as signals, we might reasonably expect a mechanism that supports the ideal strategy (i.e. the ability to ignore trivial, naturally-occurring odor fluctuations whilst retaining the capacity to discriminate subtle inter-odor differences) to be both simple and general.

One candidate for such a mechanism is generalization [1], or the ability to learn that perceptually distinct olfactory stimuli lead to common outcomes. If generalization is to support the strategy set out above, however, it would have to be the case that the extent to which animals generalize from learned odor associations to encounters with novel odor stimuli depends itself on the precise nature of the associated outcome. The experiments reported here were designed to test this hypothesis by determining whether the ability of honeybees to generalize from learned odors to novel odors is affected by changing the nature of the associated outcome. Floral scents are excellent examples of intrinsically variable natural odors: substantial variation in the ratios of the concentrations of scent compounds is observed even for flowers produced by the same plant [3], [9]–[12]. Honeybees rely heavily on scent as a signal for recognizing rewarding flowers; they adapt to rapid changes in the availability of floral resources through learning [13]. They readily discriminate odors that differ in molecular identity or concentration and can also discriminate different proportions of two odors in a mixture [14], [15]. It is the latter ability that forms the basis of our experiments: making use of an assay originally developed to study olfactory learning in honeybees [2], we trained honeybees to learn to associate fixed-proportion binary odorant mixtures with one of several outcomes, and then tested to see whether the honeybees' tendency to generalize to novel odor proportions was outcome-dependent.

## Materials and Methods

### Subjects

Worker honeybees (*Apis mellifera carnica*) were collected and restrained as described in Wright and Smith (2004) [16]. We used a total of 230 honeybees in our experiments. Each subject was fed to satiety (∼30 µL) with 1.5 M sucrose and left on the bench at room temperature for ∼24 hours before conditioning. At least 10 min before an experiment, the antenna of each subject was stimulated with a droplet of sucrose solution to provoke the proboscis extension reflex; if a subject did not respond by extending its proboscis, it was not used in the experiments.

### Odor stimuli

The odors used in our experiments were 1-hexanol and 2-octanone (99.8% purity, Sigma-Aldrich, St. Louis, MO); both of these odors are found in floral scents, are perceptually quite distinctive to honeybees [16] and have been used in several previous investigations of honeybee olfactory learning [16]–[18]. These odors were mixed as proportions from a stock solution of 2.0 M; the original, neat odorants were diluted in hexane to obtain the specific molar concentration in solution (as described previously) [16]. Odors were presented as 4 s stimuli using an apparatus described in Wright and Smith (2004) [16] at an inter-trial interval of 5 min.

### Odor ratios

To ensure that the inter-odor ratios present in our experimental stimuli compared reasonably with those present in naturally-occurring floral odor stimuli, we computed the distribution of the inter-flower ratios of the inter-odor ratios of every possible pair of ten odorant compounds sampled from a population of natural floral odors (data reported in Wright et al., 2005)[12]. The median of this ratio distribution was found to be roughly 3. Our stimulus set was prepared such that the proportions of 1-hexanol (*p_h_*) in each mixture were 0.1, 0.3, 0.5, 0.7, and 0.9. Odor pairings for the differential conditioning paradigms were intended to produce a modest, ecologically valid offset in *p_h_* and to test both ends of the *p_h_* range, so either *p_h_* = 0.1 was conditioned with *p_h_* = 0.3, or *p_h_* = 0.9 with *p_h_* = 0.7. We argue that these pairings are ecologically relevant on the grounds that when these proportions are converted into ratios, the ratio of the two most similar odor ratios experienced by the honeybees during differential conditioning is 0.9/0.1 divided by 0.7/0.3, again roughly 3.

### Conditioning

Individual worker honeybees were trained using conditioning techniques described in Bitterman et al. [2]. The relationships between odor stimuli and outcomes were determined according to one of three different reinforcement paradigms, as follows. Two of these paradigms used a differential conditioning technique [2]: in one paradigm (the + − condition), one odor was rewarded with sucrose and the other was punished with salt, whereas in the second paradigm (the + + condition) both odors were rewarded with sucrose. In a third, control paradigm (the + condition) subjects experienced non-differential conditioning (simple reinforcement): a single odor at *p_h_* = 0.1 or 0.9 was reinforced with the sucrose reward. All subjects received a total of 12 conditioning trials with an inter-trial interval of 5 min; in the two differential-conditioning paradigms, each subject received 6 trials with each of the two odor stimuli.

In the (+ −) paradigm, one odor stimulus was paired with a sucrose reward and the other odor stimulus was paired with salt punishment. The rewarded (R) and punished (P) trials were interleaved in pseudo-random order (e.g. R-P-P-R-P-R-R-P-R-P-P-R). In the (+ +) paradigm, both odor stimuli were paired with a sucrose reward and interleaved in the same pseudorandom order. In the (+) paradigm, subjects received 12 trials with the same odor rewarded with 1.5 M sucrose on each trial.

For sugar-rewarded conditioning trials, the odor stimulus was presented approximately 3 s before the delivery of a droplet of sucrose to the antenna to initiate proboscis extension; when the proboscis was extended, a 0.4 µl droplet of 1.5 M sucrose was delivered using a Gilmont micrometer syringe. Subjects were considered to have learned to associate odor with reward when they extended the proboscis in the presence of odor alone. Salt-punished conditioning trials were similar in all ways except that 1.5 M salt solution was administered to the antenna only. The testing phase, which began ten minutes after each of the conditioning paradigms, also had an inter-trial interval of 5 min; each subject received a single, unreinforced trial with each of the five different odor stimuli such that the order of presentation of the odors was randomized across subjects.

### Data pooling

Response probabilities were calculated by averaging the binary responses over all subjects for each stimulus value of the test odors. We balanced the design of the experiment such that one group of individuals were conditioned with odor stimuli at the *p_h_* = 0.1–0.3 end of the range of ratios and another group were conditioned with odor stimuli at the *p_h_* = 0.7–0.9 end of the range (Table 1) for each of the conditioning paradigms. When we compared groups conditioned at both ends of the range, we observed that the slope of the generalization gradient did not depend upon which group of odors were used as conditioned stimuli. This was true for all three conditioning paradigms ((+): χ_4_^2^ = 0.35, *P* = 0.986; (+ +): χ_4_^2^ = 1.0, *P* = 0.318; (+ −): χ_4_^2^ = 0.15, *P* = 0.699) and meant that it was possible to pool the data for both groups for each conditioning paradigm. The data reported in the results section, therefore, are these pooled data; since some subjects will have been conditioned with low-*p_h_* stimuli and others with high-*p_h_* stimuli, results are reported as a function of Δ*p_h_*, the absolute *difference* in *p_h_* between the test odors and the respective CS (i.e. either *p_h_* = 0.1 or 0.9). At least two different conditioning paradigms (e.g. the + + paradigm) were employed on any particular day in an attempt to distribute any day-to-day variation in the responses of individual subjects across all of the experimental treatments.

**Table 1:** Description of the odors used as conditioned stimuli (CS) in each different conditioning paradigm.

	CS (+)	N	CS (+ +)	N	CS (+ −)	N
Group 1	CS(+): *p_h_* = 0.1	33	CS(+): *p_h_* = 0.1 and CS(+): *p_h_* = 0.3	41	CS(+): *p_h_* = 0.1 and CS(−): *p_h_* = 0.3	27
Group 2	CS(+): *p_h_* = 0.9	27	CS(+): *p_h_* = 0.9 and CS(+): *p_h_* = 0.7	47	CS(+): *p_h_* = 0.9 and CS(−): *p_h_* = 0.7	42

Footnotes: The *p_h_* refers to the proportion of 1-hexanol present in a binary mixture of 1-hexanol and 2-octanone. The (+) represents a paradigm where a honeybee received conditioning with only one odor in association with 1.5M sucrose reward. The (+ +) paradigm represents conditioning with two odors, each associated with 1.5M sucrose. The (+ −) paradigm represents conditioning with two odors where one is associated with 1.5M sucrose and the other is associated with 1.5M salt.

### Data analyses

Both binary logistic regression and signal-detection theory were used to analyse response probabilities. Since both these techniques can be “contaminated” by inattention to the task at hand (for example, when the subject guesses the answer), the naive linearization-transformations they employ are inappropriate; to address this, we used the methodology detailed in Heinemann et al. [19] to obtain estimates of the guess rate and thereby correct the response probabilities prior to performing the analyses.

For the case of the logistic regression analyses, differences in the regression-model gradients reflect differences in the slope of the psychometric function (i.e. sensitivity) and are interpreted here as changes in generalization gradient; differences in the regression-model abscissa reflect lateral shifts in the psychometric function (i.e. bias) [20]. Dunnett's *post-hoc* multiple comparisons tests were used to compare novel-odor responses to conditioned-odor responses for each conditioning paradigm.

Our application of signal-detection theory (SDT) to these data involved treating response probabilities as performance in a ‘same-different’ discrimination task. Signal-detection theory is attractive because it provides an independent measure of sensitivity and bias in psychophysical data. This distinction is important here because it is *sensitivity* shifts that are diagnostic of changes in generalization gradient (for more information see Blough, 2001) [21]. The application of SDT to the current work assumes that the subjects in our experiments decide during the testing phase whether a stimulus is “same” or “different” to a previously experienced stimulus. SDT asserts that an observer decides between these two hypotheses by comparing the stimulus pair and forming different likelihood estimates under the “same” and “different” hypotheses. Under the assumption that these two hypotheses are represented in the relevant decision space by multivariate Gaussian probability density functions of equal variance, the raw hit rates and the false-alarm rates can be linearized by application of the inverse, cumulative Gaussian transform; their difference after linearization is the discrimination index, or *d',* of signal-detection theory. The value of *d'* is thus effectively a summary statistic of observer sensitivity, and thresholds can be estimated by specifying a criterion level of *d'* (a value of 1.0, common in the literature, was used here). Moreover, provided that the hit rate and the false alarm rate are binomially distributed across the trials, it is possible to estimate the variance of the linearized scores and hence of the *d'* itself using a bootstrapping method [22]. We employed this technique to estimate the standard deviation of the *d'* data.

## Results

**Figure 1:**
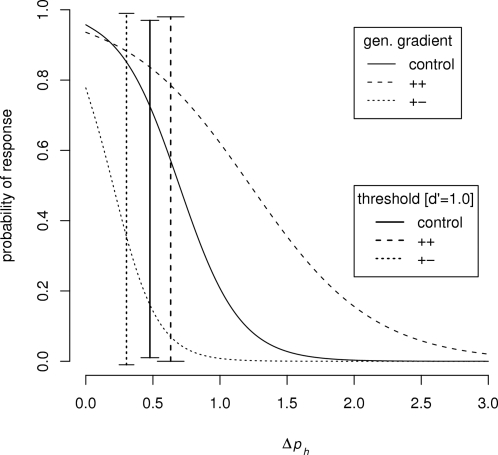
Honeybees' responses to the test odors depended upon conditioning paradigm. The filled circles represent the mean response probabilities to the test odors; the error bars indicate the standard errors predicted by a binomial response distribution. The control condition (black circles) represented a situation where honeybees received conditioning with one odor (the Δ*p_h_* = 0.0) in association with sucrose reward (N = 60). The red line represents the (+ +) condition in which a honeybee was conditioned with two odors (Δ*p_h_* = 0.0 and Δ*p_h_* = 0.2) both in association with sucrose reward (N = 98). The green line represents the (+ −) condition in which a honeybee was conditioned with the same two odors, but Δ*p_h_* = 0.0 was associated with sucrose and Δ*p_h_* = 0.2 was associated with salt punishment (N = 69).

The data presented in Figure 1 show that the honeybees' patterns of response to the test odors depended clearly on the conditioning paradigm. Pairwise comparisons of the slopes of the generalization gradients for each type of conditioning (+ vs. ++, + vs. + −, and ++ vs. + −) show that all three gradients are significantly different (see Methods; *Z* = 2.8,−3.5,−4.6; all *P*<0.05). The (+ −) condition, in which odors with a Δ*p_h_* = 0.2 are differentially reinforced, is additionally associated with a significant negative bias relative to the generalization gradients produced by the other two training conditions (+ and ++) (*Z* = −5.1,−10.0; *P*<0.05).

Within each condition, we also performed *post hoc* multiple comparisons of the responses to the CS (+) (Δ*p_h_* = 0.0) versus the responses to each of the novel test odours. For the (+ −) condition, the probability that a honeybee would respond to positively-reinforced CS (Δ*p_h_* = 0.0) was always greater than the response to all other test odor ratios (including the negatively-reinforced CS) (see Methods; *Z* = −3.5 to −6.7; all *P*<0.05). With the exception of the closest stimulus pair (Δ*p_h_* = 0.0 versus Δ*p_h_* = 0.2), this was also true for the (+) condition (*Z* = −2.5 to −5.4; *P*<0.05). Finally, the corresponding tests conducted for the (++) condition were never significantly different (*Z*< = −0.001, *P*∼1.0)).

**Figure 2:**
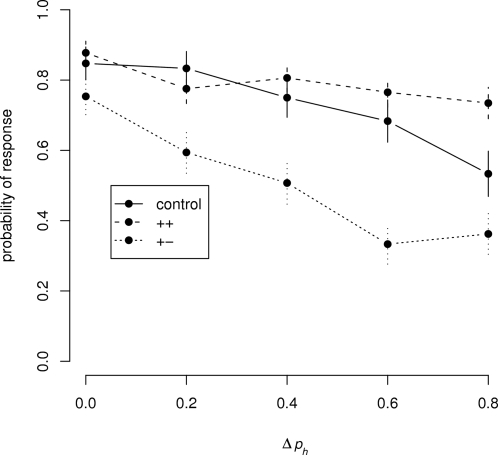
Generalization gradients for honeybees as predicted from the logistic regression models fit to the data from Figure 1. Generalization gradients for the honeybee and their relative discrimination thresholds were generated by evaluating the models fitted by the logistic regressions and extrapolating them over a wider range of Δ*p_h_* values than could be used in the experiments. The dotted lines show *d′* thresholds (see Methods) calculated for the three conditions using signal-detection theory; the horizontal bars visible at the extrema show standard deviations on these thresholds.

Applying signal-detection theory to these data (see Methods) yielded an estimated discrimination threshold of Δ*p_h_*∼0.3 for the (+ −) condition. The less selective behavior of the honeybee in the other conditions is thus consistent with the definition of generalization–the bees associate *perceptually distinct* stimuli with a common outcome– and, in accordance with previous studies of generalization [1], [16], we interpret the statistically significant changes in slope as changes in generalization gradient. These are cognitive changes arising from differences in outcome [1], [17] and they have a profound effect on the honeybees' discrimination performance. The same application of signal-detection theory also indicates that the maximum possible shift in discrimination index *d′* across conditions (++ v. + −) is similar to that across Δ*p_h_* values (0.2 v. 0.8), and that the change from (++) to (+ −) conditions roughly halves the honeybee's threshold (from Δ*p_h_* = 0.6 to 0.3) (Figure 2). Though these changes in generalization gradient are clearly outcome-mediated, they are also affected by the intrinsic variability of the conditioned stimulus, as one might expect: the change in gradient from the control to the (+ −) condition is not the same as that from the (+ +) condition to the (+ −) condition, despite the fact that conditioning stimuli were always rewarded for both the (+ +) and control paradigms. This may indicate that honeybees can effectively de-tune their sensitivity to the ratio of two odors in a mixture in situations where highly variable odors lead to a common outcome.

## Discussion

Our results imply that the honeybee has the ability to modulate its perceptual sensitivities via an outcome-mediated cognitive mechanism, since the outcome associated with a binary odor mixture strongly affected a honeybee's sensitivity to differences in the ratio of two odors. Honeybees thus have the ability to use precise information about the ratio of two odorants in a mixture in order to identify a rewarding stimulus and to discriminate it from a punishment-associated stimulus having a different ratio of the same two odorants. Alternatively, if two stimuli differing in odor ratios both lead to a rewarding outcome, honeybees can learn to ignore information about the ratio of the two odorants and thereafter respond to all mixtures of the same two odorants with equal probability.

Although our stimuli were considerably less complex than most natural odors, the experiments described here were designed such that the odorant types, odor proportions, and inter-proportion ranges used in this study are broadly concordant with data reported from headspace analyses of floral scents (see Methods). We would, therefore, contend that our experiments reflect an ecologically valid task, and predict that the ability to generalize is the means by which pollinators, and possibly other animals, are able to discount irrelevant temporal or spatial odor fluctuations in order to exploit more informative odor features.

The reliance on a cognitive mechanism to solve this problem may reflect the difficulties associated with the encoding of olfactory stimuli. Not only are there many different types of olfactory receptor involved in processing a complex olfactory scene [25], but absolute olfactory stimulus concentration also appears to contribute much more to stimulus identity than do absolute stimulus levels (e.g. luminance) in other sensory modalities [11], [16]–[18]. If odor representations do not remain invariant as a function of stimulus concentration, then changing the absolute quantities of the two odorants in a binary mixture might also change the perceptual qualities of the mixtures, and even where such perceptual changes are minor, they could still make it difficult to recognize an odor stimulus based on the molecular identities of the two odorants alone. For efficient olfactory coding to occur, invertebrates like the honeybee must implicitly not only be able to solve these problems but do so in a sensory system that lacks the remarkable functional specialization of, for example, the human neocortex. Even if it was possible for the olfactory system to extract information about an odor's molecular identity and concentration independently [26], [27], it might be too expensive computationally for such animals to do this over the entirety of their chemoreceptive range by means of hard-wired neural mechanisms. It may be necessary instead for at least some of the encoding strategies operated in other modalities by means of low-level physiological mechanisms (lateral inhibition in many visual systems, for example, maximises the stimulus signal-to-noise ratio in the face of high-amplitude stimulus fluctuations; see, e.g., Srinivasan et al., 1982) [28] to be instantiated in olfaction through a cognitive process such as generalization. Thus, in a situation where quantitative concentration fluctuations produce salient differences in qualitative properties of an odor stimulus [18], generalization could provide the means of adapting to such variation.

These arguments lead us to suggest that olfactory generalization is not merely a means of classifying similar, though perceptually distinct, stimuli (as is often concluded from studies in other sensory modalities [1], [23]); rather a mechanism used by animals to adjust their sensitivity to differences in complex olfactory stimuli in a context-dependent manner. We think it may be particularly important for animals like pollinators to deal directly with context-dependent stimulus variability because optimal odor-processing strategies must surely take direct account of the nature of the odor-outcome relationships. Honeybees, in particular, would be afforded a substantial fitness advantage: both the floral odor signals and food rewards' quality and quantity are highly variable, so the ability to modulate sensitivity to specific features in floral odors in accordance with reward quality could improve a foraging worker's efficiency for obtaining floral rewards. In situations where variability in odors was inconsequential, honeybees would be able to rapidly exploit all floral sources available, yet bees would still be able to perform subtle discriminations in situations when inter-odor differences signal significant differences in the quality of food rewards.

This strategy itself may exert selective pressure on plant-pollinator interactions, which in turn increases the complexity of the signal-outcome space. While the development of “cognition” in invertebrate pollinators is likely to have resulted from the co-evolution of complexity in and diversity of floral signals and the quality of floral rewards [29]–[31], co-evolution has also produced insect pollinators with the ability to use odor signals in a way that is more plastic than one afforded by an instinctual response. Cognitive generalization mechanisms for odor recognition afford animals the ability to avoid dishonest odor emitters–a feat largely unavailable to animals that have instinctual behavioural responses to odors. For example, male solitarious bees that are susceptible to certain sexually-deceptive orchids (e.g. *Andrena negroaena* and *Ophrys sphegodes*) can learn to avoid a specific ratio of pheromone compounds but do not appear to generalize this knowledge to other ratios of the same compounds, and so are deceived even when a flower that is located on the same inflorescence of a flower they have already visited emits different ratios of the same compounds [9]. It is likely that the fluctuation in the ratios of the “behaviourally active” pheromone compounds [32] is deliberately maintained in populations of these orchids in order to render the male bees incapable of avoiding other conspecific orchids [9]. Indeed, it is even possible that the evolution of deceptive odor signals has taken advantage of situations where instinctive responses to odor signals limit an animal's ability to detect and avoid deceivers. Animals that can maintain sensitivity to key odor-outcome relationships by learning to tune odor generalization according to outcome are at a distinct advantage at detecting potential infiltrators of olfactory signals.
