# Correction: Generalization Mediates Sensitivity to Complex Odor Features in the Honeybee

**DOI:** 10.1371/annotation/8407eea7-c39e-4c7a-82c8-f67a64175beb

**Published:** 2008-04-08

**Authors:** Geraldine A. Wright, Sonya M. Kottcamp, Mitchell G. A. Thomson

Figures 1 and 2 are displayed incorrectly. Figure 1 should be Figure 2, and vice-versa. The figure captions are correct, but the figures have been placed in the wrong order.

